# Identification of a Potent Allosteric Inhibitor of Human Protein Kinase CK2 by Bacterial Surface Display Library Screening

**DOI:** 10.3390/ph10010006

**Published:** 2017-01-05

**Authors:** Christian Nienberg, Claudia Garmann, Andreas Gratz, Andre Bollacke, Claudia Götz, Joachim Jose

**Affiliations:** 1 Institut für Pharmazeutische und Medizinische Chemie, PharmaCampus, Westfälische Wilhelms-Universität Münster, Correnstraße 48, D-48149 Münster, Germany; christian.nienberg@uni-muenster.de (C.N.); claudia.reicheneder@gmx.de (C.G.); gratz.andreas@gmail.com (A.G.); andre.bollacke@uni-muenster.de (A.B.); 2 Medizinische Biochemie und Molekularbiologie, Universität des Saarlandes, Kirrberger Str., Geb. 44, D-66421 Homburg, Germany; claudia.goetz@uks.eu

**Keywords:** autodisplay, human protein kinase CK2, non ATP-competitive inhibitor, peptide

## Abstract

Human protein kinase CK2 has emerged as promising target for the treatment of neoplastic diseases. The vast majority of kinase inhibitors known today target the ATP binding site, which is highly conserved among kinases and hence leads to limited selectivity. In order to identify non-ATP competitive inhibitors, a 12-mer peptide library of 6 × 10^5^ variants was displayed on the surface of *E. coli* by autodisplay. Screening of this peptide library on variants with affinity to CK2 was performed by fluorophore-conjugated CK2 and subsequent flow cytometry. Single cell sorting of CK2-bound *E. coli* yielded new peptide variants, which were tested on inhibition of CK2 by a CE-based assay. Peptide B2 (DCRGLIVMIKLH) was the most potent inhibitor of both, CK2 holoenzyme and the catalytic CK2α subunit (IC_50_ = 0.8 µM). Using different ATP concentrations and different substrate concentrations for IC_50_ determination, B2 was shown to be neither ATP- nor substrate competitive. By microscale thermophoresis (MST) the K_D_ value of B2 with CK2α was determined to be 2.16 µM, whereas no binding of B2 to CK2β-subunit was detectable. To our surprise, besides inhibition of enzymatic activity, B2 also disturbed the interaction of CK2α with CK2β at higher concentrations (≥25 µM).

## 1. Introduction

Human protein kinase CK2 is a heterotetrameric enzyme, consisting of two catalytic (CK2α or CK2α′) and two regulatory subunits (CK2β). The interaction between the CK2 subunits is highly dynamic and the balance between separation and interaction of the subunits to create a functional holoenzyme is crucial in the control of many cellular processes [1]. CK2 has a key role in cell signaling networks. It influences cell growth and proliferation, as numerous growth related proteins are substrates of CK2 [2]. The highly pleiotropic serine/threonine-kinase CK2 is constitutively active and present in nearly all tissues, cell types and most cell compartments. There is growing evidence for the involvement of deregulated CK2 activity in a variety of human cancers such as breast cancer [3], prostate cancer [4] and colorectal cancer [5]. Elevated CK2 activity has been associated with the malignant transformation of several tissues [6] and is supposed to serve as prognostic marker in several diseases such as acute myeloid leukemia [7] or Parkinson’s disease [8]. Therefore CK2 has emerged as a potential therapeutic target for cancer treatment [9]. Drug discovery programs were launched aiming at the identification of specific inhibitors of CK2 reducing the elevated CK2 activity in cancer cells to a non-pathogenic level. To date, several lead structures of small molecule CK2 inhibitors are known, which in nearly all cases are competitive with respect to the co-substrate ATP [10]. One relevant representative is the halogenated benzimidazole derivative 4,5,6,7-tetrabromo-benzotriazole (TBB) with a K_i_ of 0.4 µM [11]. The flavonoid quercetin (IC_50_ = 0.55 µM) and the anthraquinone emodin (K_i_ = 1.85 µM) [10] are representatives of natural compounds which show CK2 inhibition. Other potent inhibitors with IC_50_ values in the nanomolar range have a pyrazolotriazine scaffold [12] or an indoloquinazoline scaffold such as IQA (IC_50_ = 0.39 µM) [13]. To date one inhibitor was able to complete successfully phase I clinical trials. Silmitasertib (CX-4945) is an oral small molecule inhibitor of CK2, showing a selective anti-proliferative activity [14]. Currently silmitasertib is in phase II clinical trials for the treatment of cholangiocarcinoma. The high degree of conservation of the ATP binding site throughout the human kinome [2] is often a disadvantage of ATP-competitive inhibitors. As a consequence, ATP competitive kinase inhibitors, including those targeting CK2, are poorly specific and also affect other kinases. The development of inhibitors with a different mode of action promises to impact CK2 activity more selectively. The first studies revealed CK2 inhibiting compounds not directly competing with ATP. By screening of highly diverse chemical libraries it was found that inorganic polyoxometalates (POMs) are nanomolar inhibitors of the CK2 holoenzyme and CK2α subunit [15]. However the active structure of the most potent POM with an IC_50_ of 5 nM) was not identified so far. The authors speculated that POMs target an exosite of the CK2α subunit as allosteric inhibitors. Other approaches are the development of inhibitors which interfere with the assembly of the subunits. It was found that—at least in vitro—the free catalytic subunit and the holoenzyme exhibit divergent substrate preferences [16]. The disassembly and reassembly of the CK2 holoenzyme seems to be a likely point of regulation of many cellular processes [16]. An inhibitor (W16) of the CK2 subunit interaction (IC_50_ = 40 µM) was found by screening a library of podophyllotoxine indole-analogs [17]. The kinase activity of the CK2 holoenzyme was not affected by W16, but the activity of CK2α (IC_50_ = 20 µM) was inhibited. The authors assumed that W16 docks to the CK2α/CK2β interface inducing an allosteric conformational change in CK2α that affects its activity. For azonaphthalene derivatives a non-ATP competitive inhibition of both the α-subunit and the heterotetrameric CK2 holoenzyme was shown (most potent inhibitor: IC_50_ = 0.4 µM) [18]. Azonaphthalene derivates were shown to decrease tumor genesis in vitro and in vivo. The exact binding site on CK2 is not known, but it was shown that the inhibitor caused a large conformational change of CK2α, thereby blocking the binding of substrates. A different class of emerging non-ATP competitive inhibitors is comprised of peptides. Among peptides a binding at the ATP cave is unlikely, taking into account the rigid structure of ATP-competitive inhibitors, usually consisting of heterocycles [19]. The Pc peptide is a cyclic 11-mer identified by a screening of conformational constrained peptides mimicking the binding interface of CK2β with CK2α. It is able to antagonize (IC_50_ = 3 µM) and to disassemble (IC_50_ = 6.5 µM) the formation of the CK2 holoenzyme complex [20]. In contrast to W16 the Pc peptide shows no inhibition of CK2α, but it acts as CK2β antagonist, inhibiting phosphorylation of CK2β dependent substrates. Another peptide-derived inhibitor is CIGB-300. It consists of two domains, the cyclic 9-mer P15, which inhibits CK2-catalyzed phosphorylation by blocking the interaction with the substrate and the Tat-peptide for enabling membrane translocation [21]. Antitumor effects were shown, among others, on patients with cervical malignancies [22] and meanwhile CIGB-300 has also entered phase II clinical trials. Nevertheless the interaction of CIGB-300 and CK2 is not understood on a molecular level. The 18-mer peptide P1 was isolated by screening of a combinatorial peptide aptamer library. It binds to an N-terminal domain on CK2β (K_D_ = 0.4 µM) [23]. The interaction of P1 with CK2β neither dissociates the CK2 holoenzyme complex nor prevents its formation and did not inhibit the phosphorylation of two protein substrates exclusively targeted by the CK2 holoenzyme. The authors assumed that binding of P1 to the N-terminal domain of CK2β compete for a common binding site on the protein for unknown endogenous ligands or protein kinase substrates that are essential for cell survival [24]. In different mammalian cell lines apoptosis through the recruitment of a p53-dependent apoptosis pathway was induced.

**Figure 1:**
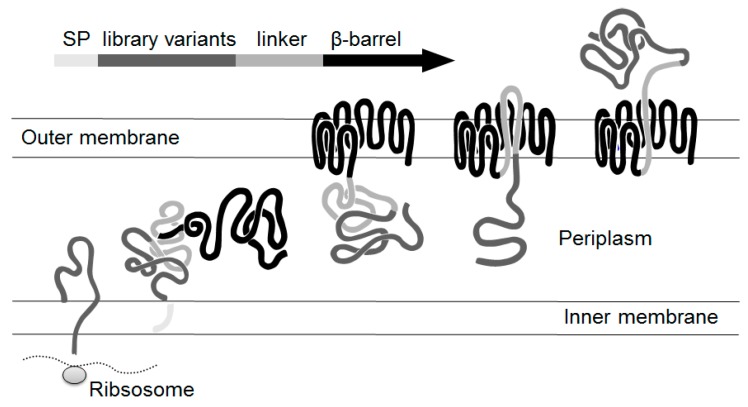
Secretion mechanism of library variants by autodisplay.The autotransporters are synthesized as precursor proteins containing all structural requirements for the transport to the cell surface. With the aid of the signal peptide, the polyprotein precursor is translocated across the inner membrane. Arriving at the periplasm, the precursor folds as a porin-like structure, the so called β-barrel within the outer membrane and the passenger is transmitted to the cell surface. To obtain full surface exposure, a linker region between the passenger and the β-barrel is required. SP: Signalpeptide.

The aim of this study was to screen a surface displayed library of peptides for a non-ATP competitive CK2 inhibitor. A major advantage of screening randomized libraries is that new structures for specific targets can be identified without needing to know the exact peptide-surface interaction. Since the invention of phage display twenty years ago, display technologies have turned out to be a successful tool for broad biotechnology applications [25,26]. By surface display of libraries on cell surfaces the variants are available for screening without a purification step. It is possible to create a high number of cells with every single cell carrying a different peptide on the surface in high numbers. After selection the sequence of the peptide or protein can be easily determined, due to the fact that every cell carries an internal label, the DNA sequence. Surface display systems have shown to be a promising tool in the development of peptide inhibitors. They were used for identification of protease inhibitors [27,28], as also for discovery of antiviral peptides [29,30]. Autodisplay which was used in this study was shown to be a flexible surface display system for presenting polypeptide libraries on the surface of *E. coli* cells [31,32,33]. In combination with flow cytometry it yielded a new lead structure for human cathepsin G inhibitors (IC_50_ = 11.7 µM) [31]. Autodisplay based transport of peptides or proteins is facilitated by the natural autotransporter secretion mechanism of Gram negative bacteria [34] (Figure 1).

In the present study a 12-mer peptide library of up to 6 × 10^5^ variants was displayed on the surface of *E. coli* cells via autodisplay. A flow cytometry-based screening based on the affinity of fluorescence coupled CK2 yielded a new allosteric inhibitor of CK2 with an IC_50_ value in the submicromolar range.

## 2. Results

### 2.1. Autodisplay of Human α_S1_-Casein and Cellular Labeling with CK2-FITC

**Figure 2:**
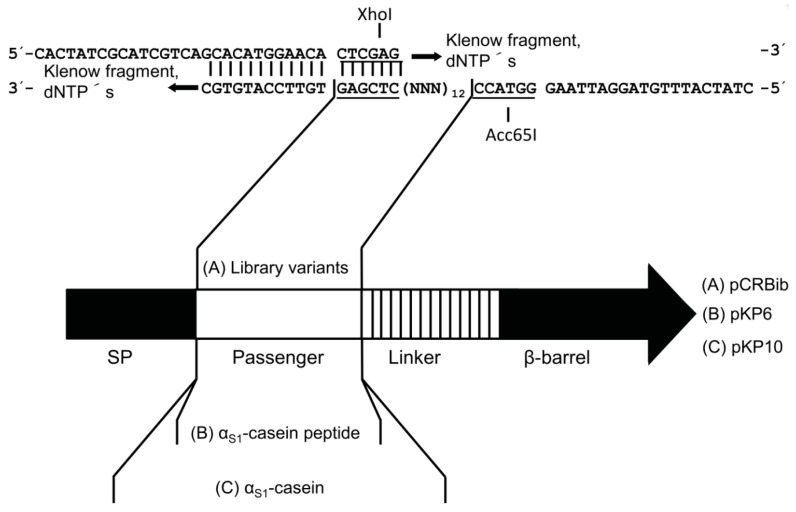
Structure of the autotransporter fusion proteins encoded by plasmid pCRBib (12mer peptide library, **A**), plasmid pKP6 (α_S1_-casein peptide, **B**) and plasmid pKP10 (α_S1_-casein, **C**) used for autodisplay. (**A**) The autodisplay passenger of plasmid pCRBib consists of 36 randomized nucleotides. The passenger region, the fusion sites to the signal peptide and the linker region of the autotransporter are given as DNA-sequences. The oligonucleotides CR21 and CR22 were hybridized and filled up to a double strand and then inserted by the underlined restriction sites. The fusion protein has a calculated molecular weight of 50.2 kDa after cleavage of the signal peptide. (**B**) Human α_S1_-casein as autodisplay passenger of plasmid pKP10 has a length of 510 nt. (**C**) The autodisplay passenger of plasmid pKP6 is α_S1_-casein peptide encoded by 48 nt. The passengers encoding sequences were inserted in frame into the autotransporter encoding gene. SP = signal peptide.

Casein was one of the first substrates reported for CK2 [35]. Previous western blot experiments with an anti-phosphoserine antibody proved that human α_S1_-casein is a substrate of CK2 (data not shown). Due to the fact that a target enzyme has affinity to its substrate it was expected that CK2 would show affinity to human α_S1_-casein. The binding of surface displayed human α_S1_-casein by fluorophore-coupled CK2 was supposed to be detectable in vitro via flow cytometry. Therefore, α_S1_-casein was inserted as passenger into an autotransporter-fusion protein and presented on the cell surface of *E. coli* (encoded by plasmid pKP10, Figure 2C).

**Figure 3:**
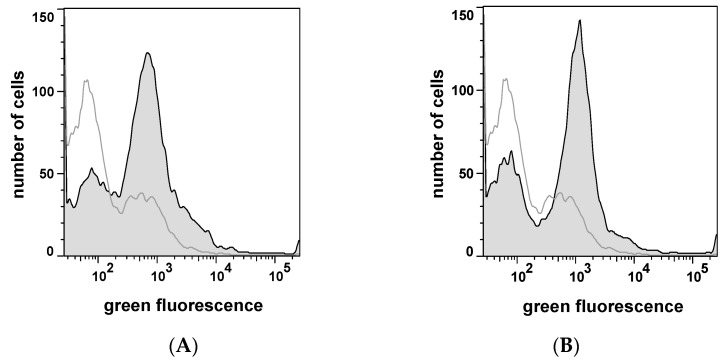
Flow cytometer analysis of *E. coli* cells displaying α_S1_-casein (170 aa) (**A**) and α_S1_-casein peptide (16 aa) (**B**) after incubation with fluorophore coupled CK2. By the use of a FACSAria flow cytometer 50,000 cells were analyzed with an exication wavelength of 488 nm and an emission wavelength between 527 and 530 nm. The host strain *E. coli* UT5600(DE3) was outlined in the same manner (grey line). (**A**): *E. coli* UT5600(DE3) pKP10; (**B**): *E. coli* UT5600(DE3) pKP6.

The surface display of α_S1_-casein was proven by protease accessibility and immunofluorescence using a polyclonal rabbit serum against α_S1_-casein [36]. The α_S1_-casein displaying cells were incubated with fluorescein-isothiocyanate (FITC) coupled CK2 holoenzyme. The amount of bound CK2 determined the overall fluorescence intensity of α_S1_-casein-displaying cells. Cells with increased fluorescence could be detected by flow cytometry indicating high affinity of CK2 to the surface presented α_S1_-casein. As can be seen in Figure 3A, cells displaying human α_S1_-casein on their surface (*E. coli* UT5600(DE3) pKP10) showed a tenfold higher fluorescence intensity than control cells (*E. coli* UT5600(DE3).

The tenfold higher amount of CK2 bound to surface displayed α_S1_-casein cells indicated the affinity of human α_S1_-casein to CK2. For the screening of a peptide library, it was indispensable to show that the affinity of CK2 to a surface-displayed peptide is detectable by flow cytometry as well. Therefore, as an additional control, a 16mer α_S1_-casein derived peptide known to bind CK2 (α_S1_-casein peptide) was surface displayed and analyzed by fluorescence labeled CK2 and flow cytometry as well (Figure 3B). For this purpose plasmid pKP6 was constructed (Figure 2B). After verification of expression and surface display (data not shown), cells of *E. coli* UT5600(DE3) pKP6 were incubated with fluorophore coupled CK2 and subjected to flow cytometry as done before for full-length α_S1_-casein. As shown in Figure 3B, α_S1_-casein peptide-displaying cells indeed had a significantly higher fluorescence than host cells without plasmid. This indicated that autodisplay of a peptide is sufficient for affinity labeling of cells by fluorophore coupled CK2.

### 2.2. Design of a Bacterial Surface Display Library

For the construction of the peptide library, a PCR primer containing a fully random sequence of 12 codons was designed (Figure 2A). A 12-mer random peptide library was chosen, because the already known peptidic inhibitors of CK2 had a length of nine, eleven and eighteen amino acids. Beyond the random sequence the oligonucleotide primer of 82 nt length (CR22) contained a 5′- as well as a 3′-extension. The 3′-entension was complementary to a shorter oligonucleotide primer (CR21), which was used to fill up the random sequence of CR22 to a double strand by Klenow fragment DNA polymerization. The 5′-extension of CR22 finally led to a double stranded DNA fragment that could be easily identified in an agarose gel, to control the reaction. The XhoI/Acc65I restriction sites flanking the random sequence were used to insert the library cassette into the open reading frame of an autotransporter artificial gene construct in plasmid pCR19 (Figure 2A). *E. coli* UT5600(DE3) electrocompetent cells were transformed with the resulting plasmid library, yielding a library size of 6 × 10^5^ colony forming units.

### 2.3. Selecting Peptides with Affinity towards CK2 by FACS

**Figure 4:**
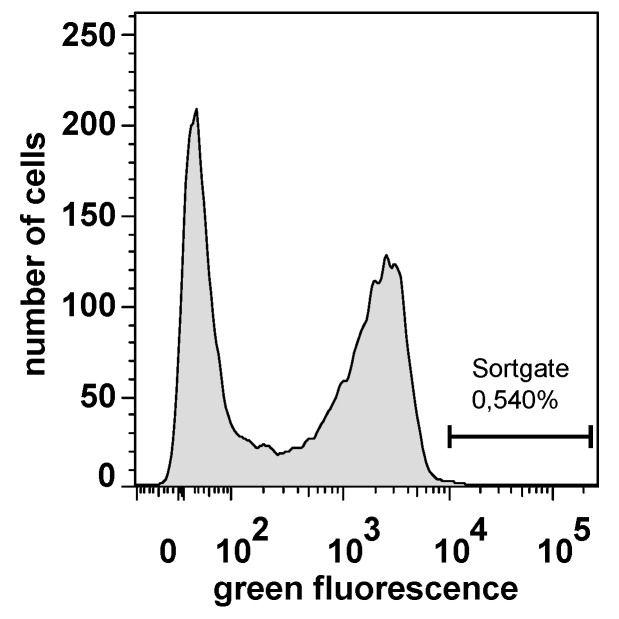
Flow cytometer analysis of *E. coli* cells displaying library peptides and sorting of cells with increased fluorescence. The analysis of 10,000 cells was accomplished with a FACSAria Cytometer with an integrated sorting module. The cells were incubated with fluorophore coupled CK2 and analyzed at a excitation wavelength of 488 nm and an emission wavelength between 527 and 530 nm. A sort gate was drawn around the population with an increased green fluorescence, which represent 0.54% of all events. These cells were selected out and grown on a agar plate for further analysis.

The *E. coli* surface display library was grown for 1 h after transformation in SOC-medium. Protein expression was induced with 1 mM IPTG. Subsequently, the complete library was incubated with fluorophore coupled CK2 and subjected to flow cytometry as described above. In the histogram plot of 10,000 cells analyzed, two populations were clearly visible, one with a strongly increased fluorescence and one with low fluorescence (Figure 4). A sorting gate was defined in order to isolate single cells with the highest values of increased fluorescence from the positive subpopulation, finally comprising 0.54% of all cells (Figure 4).

**Figure 5:**
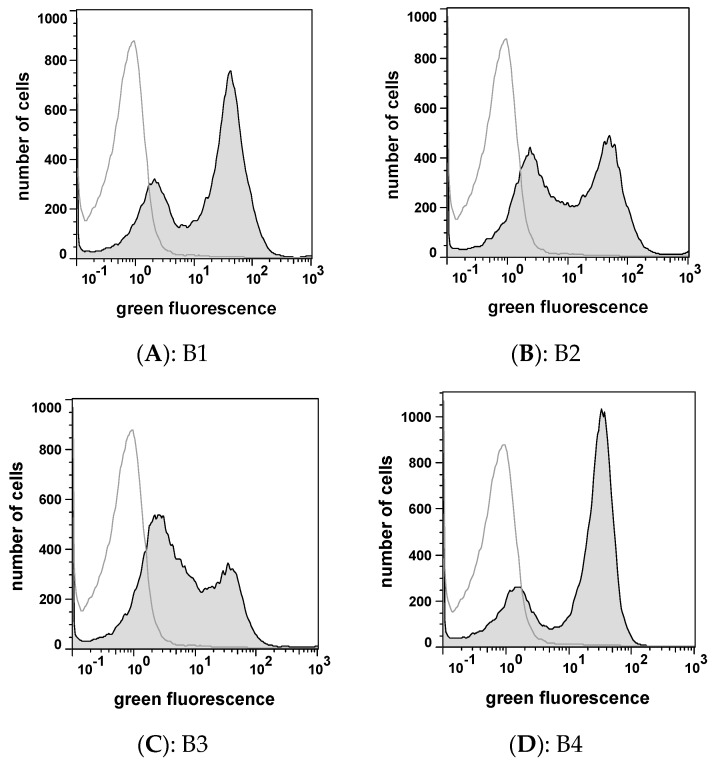
Flow cytometer based reanalysis of *E. coli* cells bearing library peptides (B1–B4) selected from the surface display library by FACS. The single cell variants, selected as single cell colonies, were incubated with fluorophore coupled CK2 as described. 50,000 cells were analyzed at a excitation wavelength of 488 nm and an emission wavelength between 527 and 530 nm by the use of a CyFlow Space (filled histograms). The host strain *E. coli* UT5600(DE3) was treated identically (unfilled histogram). (**A**): B1, *E coli* UT5600(DE3) pCR31; (**B**): B2, *E. coli* UT5600(DE3) pCR33; (**C**): B3, *E. coli* UT5600(DE3) pCR34; (**D**): B4; *E. coli* UT5600(DE3) pCR36.

Using this sort gate 22 single cell variants were placed on an agar plate for further analysis. Out of this 22 variants, six variants (B1–B6) exhibited an increased affinity to fluorophore coupled CK2 when reanalysed by flow cytometry (Figure 5), indicating that 16 single cell variants appeared to be false positives.

### 2.4. Peptide Expression and Sequence Analysis

The peptide encoding sequence of the four variants (B1–B6) was subjected to DNA sequence analysis. The derived peptides all had different aa sequences with no apparent consensus motif, however, the peptide sequence of B5 and B6 contained stop codons. After growing the cells and induction of the protein expression with IPTG, bacterial outer membrane fractions containing the autotransporter fusion proteins were isolated and analyzed by SDS-PAGE. Whereas B5 showed expression of a protein of correct size, no protein expression was detectable in variant B6. In all other samples, a protein band with a molecular weight of 50.2 kDa—as expected—was detectable. Western blot with an AIDA-I-β-barrel specific antibody labelled the same band in all samples, indicating the successful expression the peptide autotransporter fusion protein.

In outer membrane fractions of *E. coli* UT5600(DE3) pCR31 (B1), UT5600(DE3) pCR33 (B2) and UT5600(DE3) pCR36 (B4) a protein band double the size of the expected molecular weight of the fusion protein could be recognized under non-reducing conditions. These three variants contained a cysteine residue (Table 1). The dimer band disappeared when reducing conditions in the SDS-PAGE sample buffer were used and an increase of the band corresponding to the monomer of the expected fusion protein was observed (data not shown). The β-barrel of the autotransporter is flexible within the outer membrane, hence a dimerization of passengers on the cell surface is possible and has been shown by many examples before [37].

### 2.5. CK2 Inhibitor Testing by Capillary Electrophoresis (CE)-Assay

Based on the aa sequences of variants B1–B4 (Table 1), the free peptides were synthesized and tested on inhibition of CK2 holoenzyme. For this purpose a CE-based CK2 inhibition assay was used as described before [38]. For initial testing CK2 was pre-incubated with the peptide in a final concentration of 10 µM for 10 min at 37 °C. After addition of the substrate and ATP, the reaction ran for 7 min and the amount of product was determined by CE. The amount of product obtained with inhibitor was set into relation to the amount obtained with the enzyme without inhibitor. At 10 µM, two of the peptides showed no inhibition (B1 and B3), one showed a weak inhibition of 29% (B4), whereas peptide B2 could be identified as a strong CK2 inhibitor with an inhibition of 79% (Table 1).

**Figure 6:**
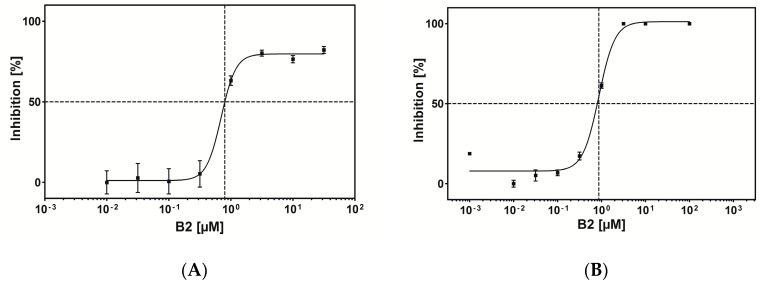
IC_50_ value determination of B2 by capillary electrophoresis measurements using CK2 holoenzyme (**A**) and the catalytic subunit CK2α (**B**). (**A**) Inhibition of recombinant CK2 holoenzyme by peptide B2 was measured after incubation with eight different compound concentrations ranging from 0.01 to 31.6 µM. (**B**) Inhibition of CK2α was tested using nine different inhibitor concentrations ranging from 0.001 µM to 100 µM. Samples were analyzed by CE. The resulting fractional inhibition values were plotted versus inhibitor concentrations in a semi-logarithmic diagram. The individual data points represent means of an experiment run in triplicates, the error bars indicate the standard deviation. In both cases an IC_50_ value of 0.8 µM was obtained by extrapolation the compound concentration at a residual CK2 activity of 50%. The dotted line marks the concentration at the midpoint (50%) of CK2 inhibition.

Apparently three of the peptides bound CK2 holoenzyme, but had no or almost no effect on enzymatic activity. For peptide B2 the IC_50_ value was determined using different concentrations ranging from 0.001 µM to 100 µM for inhibition of CK2 holoenzyme, which turned out to be 0.8 µM (Figure 6A). The IC_50_ value of B2 with the catalytic subunit CK2α alone was determined in a similar manner and turned out to be identical (0.8 µM, Figure 6B). This was a first indication that B2 interacted with CK2α directly and not via a conformational change of CK2β.

### 2.6. Mode of Inhibition

**Figure 7:**
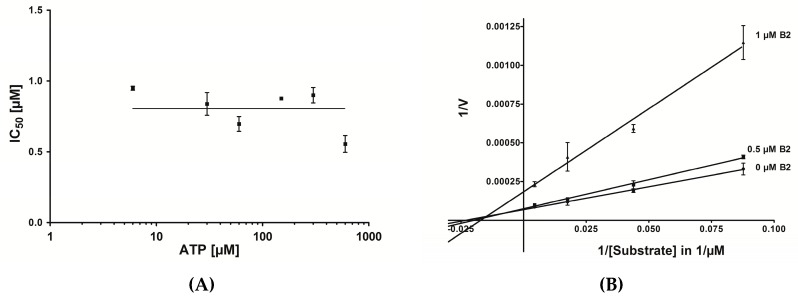
Graphical determination of the IC_50_ values of B2 in dependence of the ATP concentration and the substrate concentration. (**A**) Determination of IC_50_ values for the inhibition of CK2 by B2 with various concentrations of ATP. IC_50_ values were plotted against the corresponding ATP concentrations (ranging from 1 mM to 100 mM); the abscissa was plotted on a logarithmic scale. The individual data points represent means of an experiment run in triplicates, the error bars indicate the standard deviation. (**B**) Lineweaver-Burk inhibition plots of CK2 by B2 at different substrate concentrations. A double reciprocal plot of reaction velocity against the substrate concentration was done. CK2 kinase activity was determined in the absence (0 µM) or in the presence of 0.5 µM and 1 µM B2 with various concentration of peptide substrate. The individual data points represent means of an experiment run in triplicates, the error bars indicate the standard deviation.

In order to investigate whether B2 is competitive or non-competitive with respect to ATP the IC_50_ values were determined using six different concentrations of ATP (1 mM to 100 mM) [39]. However, as shown in Figure 7A, there was no linear increase of the IC_50_ values in dependence of the ATP concentration detectable, indicating that B2 is non-competitive with respect to ATP.

In the next step it was investigated, whether B2 could have a substrate competitive mode of action. Therefore the reaction velocity for three different inhibitor concentrations (0 µM, 0.5 µM and 1 µM B2) and varying substrate concentrations (11.4 µM, 22.8 µM, 57 µM and 228 µM peptide RRRDDDSDDD) was determined. As shown in Figure 7B, the resulting diagram with the corresponding best-fit lines intersected at a single point left from the y-axes. This is characteristic for a non-competitive mode of inhibition [39]. The conclusion was that peptide B2 is non-competitive with respect to the substrate peptide RRRDDDSDDD and non-competitive with respect to ATP. In this situation two dissociation constants can be defined, one for the binary enzyme-inhibitor complex (K_i_) and one for the ternary enzyme-substrate-inhibitor complex (αK_i_). The best-fit lines in diagram 8 nearly converge at the *x*-axis. In this case both dissociation constants are equal, hence α = 1. This means the inhibitor displays equal affinity for both, the free enzyme and the enzyme-substrate complex. According to the Cheng and Prusoff equation [40], in the case of non-competitive inhibition with α = 1, the dissociation constant is equal to the IC_50_ value (K_i_ = IC_50_) [39].

### 2.7. In Vitro Pull Down Assay

An in vitro pull down analysis with GST-tagged CK2α and in vitro translated [^35^S]-labelled CK2β was performed in order to get a hint of whether B2 could disturb the interaction of both CK2 subunits. GST or GST-CK2α were expressed in *E. coli*, purified and immobilized on a GSH Sepharose resin. The GST tag without CK2α served as a control for unspecific binding. To analyze the effect of the B2 peptide on the assembly of the subunits to a holoenzyme, the catalytic α-subunit was pre-incubated with 25 or 100 µM peptide B2 before adding the labelled CK2β-subunit.

**Figure 8:**
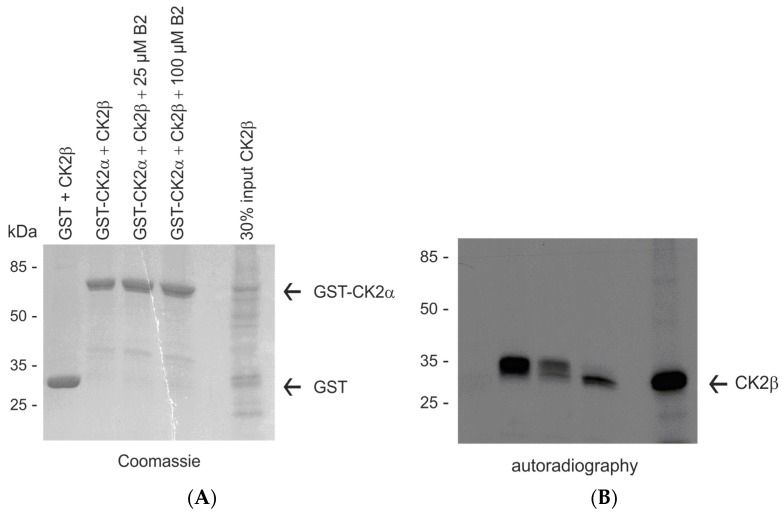
B2 interferes with the assembly of CK2α and CK2β. About 10 µg GST or GST-CK2α were incubated with 7.5 µL in vitro translated and [^35^S]methionine labelled CK2β protein in the presence or the absence of 25 or 100 µM B2 peptide, respectively. The formed complex was coupled to GSH sepharose. Proteins eluted from the affinity resins were analyzed on a 12.5% SDS polyacrylamide gel, stained with Coomassie blue (**A**) and afterwards subjected to autoradiography (**B**).

Bound proteins were eluted subsequently with sample buffer and separated by SDS polyacrylamide gel electrophoresis. The gel was stained with Coomassie brilliant blue and afterwards subjected to autoradiography to visualize [^35^S]-labelled CK2β. The Coomassie stained gel showed that equal amounts of GST-CK2α were applied in all lanes (Figure 8A). The autoradiography of the same gel also demonstrated that the GST control did not bind to the in vitro translated CK2β (Figure 8B, lane 1). GST-CK2α interacted effectively with CK2β. However, in case B2 peptide was added, the interaction was severely disturbed, resulting in significantly reduced amounts of CK2β eluted from the resin bound to GST-CK2α (compare lanes 3 and 4 with lane 2 in Figure 8B). Thus, it was concluded that the B2 peptide in concentrations of 25 µM and beyond interfered with the assembly of the holoenzyme.

### 2.8. MST Measurements

In order to support the idea that B2 binds to the catalytic CK2α subunit, the microscale thermophoresis (MST) method was applied [41]. Based on a different thermophoresis of a fluorescent protein after binding to an unlabeled interaction partner, it is possible to determine the dissociation constant (K_D_). For this approach CK2α-pAzF, which exhibited the unnatural amino acid *p*-azidophenylalanine (pAzF), was purified and coupled to the fluorophore DBCO-Sulfo-Cy5 by click chemistry as described before [42].

**Figure 9:**
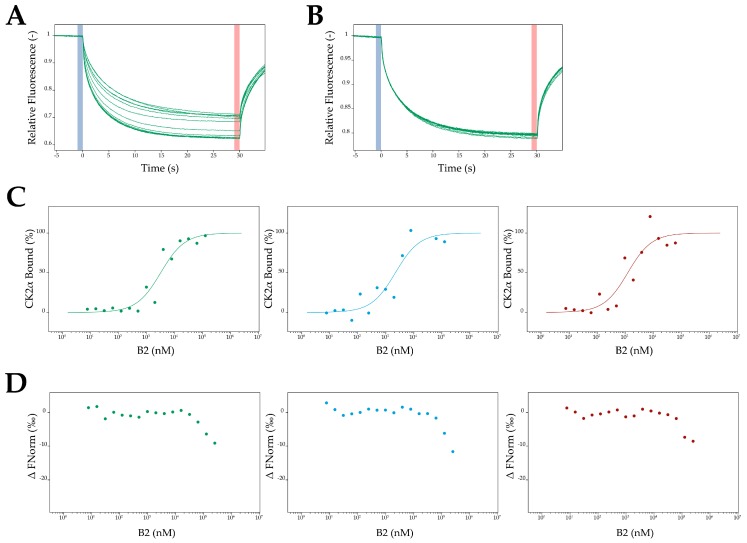
MST-measurements with B2 and CK2α or CK2β, respectively. For each subunit three independent measurements were performed and analyzed by the MO. Affinity Analysis v2.1.3 software (NanoTemper Technologies GmbH, München, Germany). (**A**) B2 was titrated in different concentrations ranging from 7.6 nM to 125 µM to a constant amount of CK2α (100 nM). The relative fluorescence signals of the thermophoresis of 15 different dilutions of B2 were recorded. (**B**) To a constant amount of CK2β (100 nM) concentrations of B2 ranging from 7.6 nM to 250 µM were added. A change in the relative fluorescence signals of the thermophoresis could only be detected in higher concentrations (above 62.5 µM). (**C**) The change in the thermophoresis led to a sigmoidal plot based on an unbound and a bound state of CK2α. This was done in three independent replicates. A mean K_D_ value of 2.16 ± 0.79 µM was determined. (**D**) The normalized fluorescence signals of the thermophoresis of CK2β^1-193^-Flu were plotted against the concentrations of B2 in three independent replicates.

Afterwards, different concentrations of B2 ranging from 7.6 nM to 125 μM were titrated to a constant amount of CK2α-DBCO-Sulfo-Cy5 (100 nM). A concentration dependent change in thermophoresis of CK2α was observed, which clearly indicated binding of B2 to CK2α (Figure 9A). The sigmoidal plot obtained by the differences in thermophoresis of the unbound state and the B2 bound state of CK2α was used to determine the K_D_ value, which turned out to be 2.16 ± 0.79 µM (Figure 9C). This was in good accordance with the inhibition of B2, either with CK2α or heterotetrameric CK2 holoenzyme (α_2_β_2_). As a control the interaction between the regulatory CK2β subunit and B2 was investigated by MST. For this purpose a truncated version of CK2β (CK2β^1–193^) was applied [43] and the unnatural amino acid pAzF was incorporated in CK2β^1–193^ at position 174 instead of tyrosine, based on the method of Chin et al. [44]. Purification of CK2β^1–193^-pAzF was performed according to the purification of CK2α-pAzF [42] and subsequently CK2β^1–193^-pAzF was coupled to fluorescein (Flu) by click chemistry. B2 was added in concentrations ranging from 7.6 nM to 250 µM to constant a concentration of CK2β^1–193^-Flu (100 nM) and applied to MST measurements. Only in very high concentrations (beyond 62.5 µM) a change in thermophoresis was detectable (Figure 9B,D). The determination of a K_D_ value was not possible by this strategy, and a K_D_ value, if any at all would be higher than 100 µM. In consequence the MST measurements clearly indicated the binding of B2 to CK2α and not to CK2β.

## 3. Discussion

By surface display library screening a potent peptidic inhibitor of CK2 holoenzyme and the α-subunit was identified. It was the 12-mer peptide B2, with the amino acid sequence DCRGLIVMIKLH. By a blast search in the UniProt protein database, no protein containing a similar sequence could be identified. B2 inhibited the catalytic activity of the α-subunit and the CK2 holoenzyme with an identical IC_50_-value of 0.8 µM. By microscale thermophoresis measurements it was possible to determine the K_D_ value of B2 with the catalytic α-subunit experimentally and it turned out to be in the same order of magnitude. At higher concentrations beyond 25 µM, the peptide also disturbed the interaction of the catalytic α-subunit with the β-subunit of CK2 to form the holoenzyme. The question arises whether these two effects are facilitated by binding to the same allosteric binding site or by binding of two different sites at the α-subunit. Comparison of the dose-response curves used for the IC_50_-value determination with the α-subunit and the holoenzyme (Figure 6A,B) reveals that—despite identical IC_50_ values—complete inhibition was only obtained with the α-subunit, but not with the holoenzyme (max. 80% for the highest concentration of B2). This could be seen as an indication for a second binding site. Nevertheless, co-crystallization experiments of B2 with the α-subunit would help answering this question and are on the way at current.

The first step in the development of this new inhibitor was the surface display of a random 12-mer peptide library on the cell surface of *E. coli* by autodisplay. Beside the well-established phage-display, surface display on bacteria and yeast has emerged to a promising tool in drug discovery [45]. One advantage of bacteria in comparison to phage particles is that they are a big enough for flow cytometry analysis. The combination of library display on *E. coli* and subsequent fluorescence activated cell sorting is a powerful and rapid method, successfully applied in the development of new antibodies [46,47,48] and in the identification of high affinity peptides for different target proteins [49,50]. In this study the strategy for identifying new non-ATP competitive inhibitors of CK2 based on autodisplay, as autodisplay has been proven before suitable for the identification of new inhibitors of drug targets [31]. With the presented strategy, it was possible to create surface-displayed peptide libraries with up to 6 × 10^5^ cell variants. After incubation with fluorescence-coupled CK2 the library could be directly used for a screening with whole cells by fluorescence activated cell sorting by which cells with increased green fluorescence were selected. After sorting, the coding sequences of the peptides presented on the cell surface could be easily determined by DNA sequence analyses of the co-selected plasmids. Within the selected variants, six peptide sequences were identified and four were tested on inhibition of the kinase activity of CK2 holoenzyme. Peptide B2 is a potent inhibitor of CK2 with a K_i_ value of 0.8 µM. The other peptides showed no inhibition or only weak inhibition. This was expectable, because the peptides were selected by their affinities towards CK2. Peptides binding to the target enzyme without influencing the activity of the enzyme can be selected by this way as well. Not expected was the high number of false positives selected by this strategy. False positives in this sense means that cells forming colonies on the agar plates after FACS selection, turned out to be negative after growing and re-labelling with CK2. This could have been due to several reasons and needs optimization. For example, a positive and a negative cell could have passed the laser beam in the detector so close that discrimination between the two was not possible, and both would have been selected. A mutant variant, not displaying any peptide could have been selected in one event with an active variant, and could have overgrown the peptide bearing strain during re-culturing. It could have been possible as well, that a peptide mutant selected had some extent of toxicity for the cells displaying it, again resulting in an overgrowing by cells not displaying it anymore. This will require systematic investigation and optimization, using mixtures of positive and negative variants with statistical analysis.

The ATP binding site is highly conserved throughout the human kinome. Thus most of the known ATP competitive inhibitors of CK2 exhibit a low selectivity across human protein kinases. By choosing a library of peptides, the development of a non-ATP competitive inhibitor was anticipated expecting a higher selectivity. Up to now three peptides interacting with CK2 are known, two of them are cyclic peptides, the Pc—peptide consist of 11 amino acids [20] and P15 is a 9-mer peptide [51] and one peptide (P1) which consists of 19 amino acids [23]. They all act as non-ATP competitive inhibitors thereby each showing a different mode of inhibition and inhibitory potency. Due to the fact that in the present study an inhibitor of the CK2 holoenzyme was identified, a comparison of effects of B2 to other described peptides with inhibitory activity towards CK2 is difficult. In the case of peptide P15 there are no in vitro data existing about the inhibition of the CK2 holoenzyme catalyzed phosphorylation of substrates [38] and it is not known if the inhibition is mediated by an interaction of the peptide with the CK2 or other effects in the cells. The Pc-peptide just inhibits the phosphorylation of CK2β-dependent substrates [17] and the peptide P1 shows no inhibition of CK2 holoenzyme activity at all [20]. In this study for the first time a potent peptidic inhibitor of the CK2 holoenzyme could be identified. The identified inhibitor B2 with a Ki value of 0.8 µM is the most potent peptidic non-ATP competitive inhibitor of CK2 known until now. Peptide B2 also inhibits the kinase activity of the alpha subunit with an IC_50_-value of 0.8 µM, thus we concluded that B2 targets the alpha subunit. CK2 exhibits different exosites distinct from the catalytic cavity that may be targeted by small molecules [52]. Based on the fact that B2 inhibits the CK2 holoenzyme and the alpha subunit with the same IC_50_ value, it is rather unlikely that B2 targets the subunit interface in or near the CK2β binding pocket on CK2α. The existence of two different classes of compounds targeting the CKα/CKβ interface is described [53]. For the podophyllotoxine W16, targeting the CKα/β interface, an inhibition of the alpha subunit but no inhibition of the holoenzyme was observed. The authors assumed that unlike CK2β, docking of W16 into this binding site might induce an allosteric conformational change of CK2α thereby blocking a productive binding of substrates [17]. The Pc peptide is also targeting the CK2α/CK2β interface, but showing another mode of inhibition. It acts as CK2β antagonist thereby inhibiting the phosphorylation of CK2β dependent substrates by the holoenzyme [20]. Thus the Pc peptide is not influencing the catalytic activity of CK2α. By sequence comparison of B2 with Pc, a similarity of less than 30% was obtained. Based on these results it can be concluded, that B2 is belonging to neither of the two classes. It is more likely that B2 binds to an allosteric cavity of CK2α, thereby changing its conformation resulting in a decrease in activity of CK2α. This mode of inhibition was already described for azonaphthalene derivatives [18] and POMS [15], both small-molecule inhibitors of CK2. Azonaphthalene derivatives act as non-ATP competitive inhibitors, affecting the kinase activity of CK2 holoenzyme and the alpha subunit in each case with the same IC_50_ value (0.4 µM). The exact binding site of azonaphthalene derivatives is still unknown, but mutation analysis indicated that they induce a conformational change of CK2α, resulting in a non-productive binding of substrates. POMs inhibit the kinase activity of CK2 holoenzyme and alpha subunit in the nanomolar range. The authors identified inhibitor-interacting domains consisting of key structural elements of CK2α, like the activation segment. They assume that by binding of the inhibitor the alpha subunit is locked in an inactive conformation. Regarding the results for the binding studies performed for azonaphthalene derivatives and POMs it can be hypothesized, that B2 docks to a similar exosite of CK2α. With B2 the first peptide could be identified which is supposed to bind to an allosteric binding site of CK2α, thereby showing a potent inhibition of the activity of catalytic CKα and also of the CK2 holoenzyme.

## 4. Materials and Methods

### 4.1. Chemicals and Reagents

Glycerol, sodium dodecyl sulfate (SDS), Tris, skimmed milk powder, Tween 20, tryptone, yeast extract and isopropyl β-d-1-thiogalactopyranoside (IPTG) were purchased from Roth (Karlsruhe, Germany), bromphenol blue, NaCl and acetic acid from Fisher Scientific (Schwerte, Germany), Coomassie brilliant blue R250 from Serva (Heidelberg, Germany), MgCl_2_, NaOH, kanamycin and carbenicillin from Fluka (Buchs, Switzerland), polyvinylidene difluoride membranes from Schleicher & Schuell (Dassel, Germany) and 1,4-dithiothreitol and goat-anti-rabbit IgG secondary antibody (10.5 mg/mL) from Sigma (Deisenhofen, Germany). AIDA-I-β-barrel specific immune serum was purchased from Davids Biotechnology (Freiburg, Germany), PageRuler™ Prestained Protein Ladder and Klenow fragment from Fermentas (St. Leon-Rot, Germany), Mung-Bean-Nuclease, XhoI and KpnI from New England Biolabs (Frankfurt, Germany). Plasmid pET-11d and pCOLA-duet1^TM^ were purchased from Merck KGaA (Darmstadt, Germany). Primers RM2, KP9, CR17 and CR18 were purchased from Sigma Aldrich (St. Louis, MO, USA) and primers CR21 and CR22 from Eurofins MWG Operon (Ebersberg, Germany). Peptides B1, B2, B3 and B4 for capillary electrophoresis (CE) measurements were ordered at JPT (Berlin, Germany). CK2 substrate peptide (RRRDDDSDDD) was synthesized by the biological medicinal research center Düsseldorf (Germany). Peptides had a purity >90% and were analyzed by HPLC & MS. The peptides delivered freeze dried and were resuspended in DMSO to a final concentration of 10 mM, subsequently they were stored at −20 °C. Human CK2α was prepared as described before [54].

### 4.2. Bacteria, Plasmids, and Culture Conditions

*E. coli* strain UT5600(DE3) [Fˉ, ara-14, leuB6, secA6, lacY1, proC14, tsx-67, ∆(ompT-fepC)266, entA403, trpE38, rfbD1, rpsL109(Str), xyl-5, mtl-1, thi-1, λ(DE3)] was used for surface display of the peptide library, of α_S1_-casein and the N-terminal shortened sequence of α_S1_-casein (α_S1_-casein peptide). *E. coli* strain BL21(D3) [B, Fˉ, dcm, ompT, lon, hsdS(r_B_ˉ m_B_ˉ), gal, λ(DE3)] was used for the recombinant expression of human CK2 holoenzyme. Plasmid pET-Adx encodes all AIDA-I autotransporter domains needed for surface display as described for plasmid pET-SH7 [55] and directs Adrenodoxin [37], the passenger domain to the cell surface. The backbone is based on pET-11d. Plasmid pKP10 was used for the autodisplay of α_S1_-casein [36]. The autotransporter region of plasmid pCR3 derived from plasmid pET-Adx, the passenger was exchanged against the coding region for MS-S1 [56] and the backbone is based on pCOLA-duet1^TM^.

The design and construction plasmid pCK2α^Y239Stop^ encoding for CK2α-pAzF was described before [42]. For pCK2β^1-193,Y176Stop^, the plasmid pT7-7CK2β was used as template in PCR. Site directed mutagenesis was performed by the use of the QuickChange protocol (Stratagene, San Diego, CA, USA). For the truncation of CK2β to CK2β^1–193^ in order to avoid aggregation problems usually obtained with full length CK2β, the primers 5′-G-CTG-GTA-GGC-CAT-**TTA**-ATG-GAT-CTT-GAA-ACC-G-3′ and 5′-C-GGT-TTC-AAG-ATC-CAT-**TAA-**ATG-GCC-TAC-CAG-C-3′ were used (mutation in bold). For the incorporation of the stop codon at position 174 the primers 5′-GG-TCT-CTT-GGG-CCG-**CTA-**CTC-GGG-ATG-CAC-CAT-G-3′ and 5′-C-ATG-GTG-CAT-CCC-GAG-**TAG-**CGG-CCC-AAG-AGA-CC-3′ were applied (mutations in bold). Plasmid pCK2β^1-193,Y176Stop^ as obtained after both sites directed mutagenesis was verified by DNA-sequence analysis (Seqlab, Göttingen, Germany). The plasmid pEVOL-pAzF (Addgene plasmid #31186) [44], which was used for the incorporation of the unnatural amino acid pAzF, was a gift from Peter G. Schultz (The Scripps Research Institute, La Jolla, CA, USA).

Bacteria were routinely cultivated at 37 °C in lysogeny broth (LB medium, 10 g tryptone, 5 g yeast extract, and 10 g NaCl per liter) with vigorous shaking (200 rpm). Depending on the vector backbone, the medium contained 30 mg/L kanamycin or 100 mg/L carbenicillin.

For flow cytometer experiments and outer membrane isolation cells were grown overnight and diluted 1:1000 in a freshly prepared medium. Cells were cultured at 37 °C with shaking at 200 rpm until the optical density (OD_578_) reached 0.5–0.7. Protein expression was induced by the addition of 1 mM IPTG and subsequent incubation for 1 h at 30 °C with vigorous shaking (200 rpm).

### 4.3. Plasmid Construction Using Restriction Sites

For the construction of the plasmid directing α_S1_-casein peptide to the cell surface the autotransporter framework of pET-Adx was used. The passenger was replaced by a 48 bp fragment coding for the α_S1_-casein peptide. Plasmid pHaS1C2 [57] was amplified with primers KP9 (5′-GCC-TCG-AGC-CCA-CTG-CTC-ATG-AAA-ATT-ATG-3′) and RM2 (5′-CGG-TAC-CCC-ACT-GTA-GCA-TGA-CG-3′) which contained overlapping ends suitable for an insertion in a XhoI/KpnI digested pET-SH7 vector, the resulting plasmid was named pKP6.

### 4.4. Synthesis of Double Stranded DNA (dsDNA)

For generating artificial dsDNA two oligonucleotides were synthesized. The oligonucleotides CR17 (5′-CAT-TCC-ATG-GTT-AAA-TTA-AAA-TTT-GGT-GTT-TTT-TTT-ACA-GTT-TTA-CTA-TC-3′) and CR18 (5′-TGC-CCT-CGA-GTG-TTC-CAT-GTG-CAT-ATG-CTG-AAG-ATA-GTA-AAA-CTG-TAA-AAA-AAA-C-3′) were used for the construction of a shortened sequence between the restriction site of the signal peptide and the passenger. For creation of the library CR21 (5′-GCA-CTA-TCG-CAT-CGT-CAG-CAC-ATG-GAA-CAC-TCG-AG-3′) and CR22 (5′-CTA-TCA-TTT-GTA-GGA-TTA-AGG-GTA-CC-(NNN)_12-_CTC-GAG-TGT-TCC-ATG-TGC-TG-3′) were used. The oligonucleotides were rehydrated (each 100 µM in water), mixed and hybridized by incubation at 95 °C for 5 min, followed by a cooling down to 36 °C within 10 min. Hydrogen bonds between 23 bp (CR17 and CR18) respectively 20 bp (CR21 and CR22) at the 3′-ends of the oligonucleotides were built. The overhanging ends were filled up with Klenow fragment and the resulting dsDNA was digested with NcoI/XhoI (CR17 and CR18) or by XhoI and Acc65I (CR21 and CR22).

### 4.5. Construction of the Plasmid Used for Surface Display of the Library

Plasmid pCR3 contained all structural elements for autodisplay. In order to remove seven dispensable amino acids between the cutting site of the signal peptidase and the passenger of plasmid pCR3, we used primers CR17 and CR18, which coded for the shortened sequence between a NcoI and a XhoI restriction site. In order to generate this artificial dsDNA, they were treated as described above and inserted into a NcoI/XhoI digested pCR3 vector. The resulting plasmid was named pCR13. The backbone of this plasmid is derived from pCOLA-duet1^TM^. In the second multiple cloning site, the Acc65I restriction site was deleted by a restriction digest with BlpI/NotI, treatment with Mung-Bean-Nuclease and religation of the vector. The new created plasmid was named pCR18. The passenger DNA of pCR18, which could be replaced by the DNA encoding the library was too short (36 bp) to be analyzed by agarose gel electrophoresis before the following cloning reaction. In consequence it was replaced by the larger passenger sequence encoding for Adx (394 bp) by the use of the XhoI and Acc65I restriction site [36]. The resulting plasmid was named pCR19, which served as backbone for the peptide library.

### 4.6. Ligation

The ligation reaction contained T4-DNA ligase (1 Weiss unit/10 µL), the DNA fragments which should be ligated and 1 × T4-DNA-ligase-puffer in water. The vector to insert molar ratio was 1:3 in a volume of 10 µL, thereby comprising 0.25 ng DNA. In the case of a ligation in library construction a vector: insert ratio of 7:1 was used in a volume of 90 µL, thereby comprising 2.5 µg DNA. The ligation reaction was incubated for 16 h at 16 °C and the reaction stopped by incubation at 65 °C for 10 min. The product of ligation was desalted via dialyses and used for transformation of electrocompetent *E. coli* strains. The ligation mixture of a library transformation was concentrated to a volume of 20 µL by the use of a rotational vacuum concentrator prior to transformation of cells.

### 4.7. Transformation of E. coli by Electroporation

Fifty µL of electrocompetent cells (2–3 × 10^10^ cells/mL) were used for transformation of 10 µL ligation product. The full reaction was placed into a chilled electroporation cuvette and exposed to a voltage of 1800 V by the use of electroporator 22510 (Eppendorf, Hamburg, Germany). Immediately after 1 mL of preheated SOC media (20 g/L Trypton, 5 g/L, Yeast extract, 0.5 g/L NaCl, 2.5 mM/L KCl, 20 mM d-Glucose, 10 mM MgCl_2_) was added and the reaction was carried into a sterile micro reaction tube. Subsequently the transformation reaction was incubated for 60 min at 37 °C and 100 rpm. Respectively 1:50, 1:500 and 1:5000 dilutions in a volume of 100 µL LB media were platted on antibiotics containing agar plates for estimation of transformation efficiency.

### 4.8. Outer Membrane Preparation

Differential cell fractionation was performed according to the rapid isolation method of Hantke [58] using modifications by Schultheiss et al. [59] without resuspending in *N*-Lauryl sarcosine sodium salt and the following centrifugation step.

### 4.9. SDS-PAGE and Western Blot Analysis

For SDS-PAGE outer membrane preparations were diluted with sample buffer (100 mM Tris/HCl pH 6.8, 4% SDS, 0.2% bromphenol blue, 20% glycerol). If reducing conditions were used additional dithiothreitol (0.2 M) was added. Samples were boiled for 10 min, analyzed by SDS-PAGE and the proteins were visualized with Coomassie brilliant blue R250 staining. Prestained protein ladder was used to determine the apparent molecular weight of the separated proteins. For western blot analysis gels were electroblotted to polyvinylidene difluoride membranes and were blocked in TBS with 3% skimmed milk powder for 1 h. Membranes were incubated with the AIDA-I-β-barrel specific immune serum, diluted 1:500 in TBS with 3% skimmed milk powder, overnight at 4 °C. The blots were washed three times with TBST, the secondary antibody (10.5 mg/mL horseradish peroxidase linked goat-anti-rabbit IgG secondary antibody, diluted 1:10,000 in TBS with 3% dried milk powder) was added, and the blots were incubated for 1 h at room temperature. Proteins were visualized via chemiluminescence.

### 4.10. Preparation of Recombinant Human Protein Kinase CK2

Recombinant human CK2 holoenzyme was expressed and purified according to a protocol by Grankowski et al. [54] with modifications described in Gratz et al. [38]. CK2 activity was determined by radiometric filter assay as described earlier [60,61]. This protocol resulted in an amount of 50 mg CK2 holoenzyme with a concentration of 1 mg/mL. Fractions containing active CK2 were pooled and stored in aliquots at −70 °C.

### 4.11. Coupling of CK2 with Fluorescein Isothiocyanate (FITC)

Purified CK2 holoenzyme (1 mg/mL) was covalently coupled to 5(6)-fluorescein isothiocyanate (FITC) using the “FITC labeling Kit” from Calbiochem (San Diego, CA, USA) according to the instructions provided by the manufacturer. Before the labeling reaction CK2 solution was dialyzed against carbonate buffer which is recommended for the labeling reaction. The FITC labeled CK2 was stored in aliquots in PBS puffer with sodium azide (0.1%) at −20 °C.

### 4.12. Flow Cytometer Analysis and Sorting

Cells were harvested and washed three times with reaction buffer (50 mM Tris/HCl, pH 7.4, 100 mM NaCl, 10 mM MgCl_2_, 1 mM DTT) and resuspended in reaction buffer with 200 µm ATP to a final OD_578_ of 1. Subsequently, 40 µL of this solution were incubated with 10 µL Fluorescein-coupled CK2 (1 mg/mL) for 30 min at 37 °C with exclusion of light. After adding reaction buffer (with 200 µM ATP) to a final volume of 500 µL, an additionally incubation for 5 min at 37 °C occurred. Accordingly cells were sedimented and washed three times with 200 µL reaction buffer (with 200 µM ATP) and resuspended in 200 µL of this buffer for flow cytometer analysis. For each experiment at least 10,000 cells were analyzed with a FACSAria III instrument (Becton-Dickinson, Heidelberg, Germany) with a ACDV option for sorting experiments or with a CyFlow Space (Partec, Münster, Germany) for analysis. In all cases an excitation wavelength of 488 nm and a 527/30 nm filter for monitoring fluorescence was used. Cytometry data were visualized by FlowJow 9.4.10 (Tree Star, Ashland, OR, USA). In the case of sorting, single bacteria cells were deposited to LB agar plates containing 30 mg/L kanamycin and were grown over night.

### 4.13. Capillary Electrophoresis (CE)-based CK2 Assay

The inhibitory activity of the selected peptides on the CK2 holoenzyme and the CK2α subunit was determined using a non-radiometric capillary electrophoresis (CE) assay of Gratz et al. [38] with slight modifications. As substrate the common CK2 peptide RRRDDDSDDD was used. In the case of CK2α the amount of NaCl in the kinase and assay buffer was reduced to 20 mM, since the CK2α subunit without β is salt sensitive. The incubation time of CK2 holoenzyme reaction was reduced to 7 min, to keep the reaction within the linear range. Due to the reduced activity of the CK2α subunit and the different temperature optimum, the reaction of CKα was performed at 25 °C for 30 min. A ProteomeLab PA800 System (Beckman, Coulter, Krefeld, Germany) was used for separation. Detection of the phosphorylated and the unphosphorylated peptide was possible by the absorption maximum of the peptide (195 nm) measured by its shoulder at 204 nm. The 32 karat 9.1 software (Beckman Coulter) served for operating the measurement and analyzing the results.

For determination of IC_50_ values a concentration-response analysis of the compound of interest was performed as described in Gratz et al. [38]. Concentrations ranging from 0.001 to 100 µM were used. The calculation of the date occurs by GraphPad Prism 5 (GraphPad, La Jolla, CA, USA). The IC_50_ value represents the concentration at the midpoint (50%) of CK2 inhibition on a semi logarithmic dose-response plot.

### 4.14. Mode of Inhibition

To figure out the mode of inhibition the previously described CK2 assay was used. For testing if the inhibitor is competitive with respect to ATP, IC_50_ values were estimated as described before and plotted versus the ATP concentration. Thereby measurements were performed using six different ATP concentrations, ranging from 1 mM to 100 mM.

For testing if the inhibitor is competitive with respect to the substrate peptide, the rate of the substrate peak at three different inhibitor concentrations (0 µM, 0.5 µM and 1 µM) were measured. The reaction time of CK2 was reduced from 7 to 6 min, so the reaction is within the linear range for substrate concentrations, which are lower than under standard conditions (114 µM). These data were plotted in a Lineweaver-Burk diagram against four varied substrate concentrations (11.4 µM, 22.8 µM, 57 µM and 228 µM) [39].

### 4.15. Expression and Purification of GST-CK2α

The cDNA of human CK2α was cloned in frame in the BamH1 site of pGEX4-T-1 (GE Healthcare, Freiburg, Germany). GST-CK2α was expressed in *E. coli* XL1Blue according to the protocol of Aberle et al. [62]. Bacteria were harvested by centrifugation at 5000× *g* and 4 °C for 10 min. The bacterial pellet from 500 mL culture was resuspended in 25 ml buffer R1 (100 mM Tris-HCl, pH 7.8, 100 mM NaCl, 10 mM MgCl_2_, 0.1% Tween 20) supplemented with protease inhibitor (Complete^®^, Roche Diagnostics, Mannheim, Germany) and 1 mg/mL lysozyme. Cells were lysed on ice by stirring for 15 min, followed by subsequent sonification. After centrifugation at 20,000× *g* and 4 °C for 10 min, the supernatant was subjected to affinity purification with 300 µL glutathione sepharose beads (GE Healthcare) pre-equilibrated with cold Buffer R1. The cell lysate was incubated with the beads under slight agitation at 4 °C for 90 min. The resin was washed with at least 30 beads volumes of Buffer R1. For pull-down experiments immobilized GST-fusion protein was left on the resin.

### 4.16. In Vitro Translation of CK2β

The cDNA of human CK2β in a pRSET A plasmid [63] was used for the T7-polymerase dependent in vitro translation by a reticulocyte lysate. CK2β was in vitro translated in the presence of [^35^S]methionine according to the manufacturer´s recommendations (TNT T7 coupled reticulocyte lysate system, Promega GmbH, Mannheim, Germany).

### 4.17. Pull Down Assay with Recombinant Proteins

The pull down assay was essentially done as described by Sun et al. [64]. Purified GST or GST-CK2α (10 μg) were immobilized on GSH-sepharose and equilibrated with PBS-T binding buffer (PBS, pH 7.4, 1% Tween 20). Immobilized proteins were pre-incubated with 25 or 100 µM B2 peptide for 1 h at 4 °C under slight agitation. Then 7.5 µL of in vitro translated CK2β was added to the mixture and incubation was continued over night at 4 °C. After washing three times with cold PBS-T, bound proteins were eluted with SDS sample buffer (65 mM Tris-HCl, pH 6.8, 2% SDS, 5% β-mercaptoethanol, 10% glycerol, 0.01% bromophenol blue) and analysed by SDS polyacrylamide gel electrophoresis, followed by protein staining with Coomassie blue and autoradiography.

### 4.18. Click Reaction of CK2α-pAzF and CK2β^1-193^-pAzF

The biosynthesis and purification of CK2α-pAzF was performed as described before [42]. For the recombinant expression of CK2β^1-193^-pAzF, plasmids pCK2β^1-193,Y176Stop^ and pEVOL-pAzF were used to transform *E. coli* BL21(DE3). CK2β^1-193^-pAzF was obtained and purified referring to the process of CK2α-pAzF [42] with the exception that before purification the cell lysate was stirred overnight at 4 °C in order to extract CK2β^1-193^-pAzF [54]. 

Purified CK2α-pAzF (130 µg/mL) in buffer P50 (25 mM Tris/HCl (pH 8.5), 50 mM NaCl) was incubated with 50 µM DBCO-Sulfo-Cy5 (Jena Bioscience, Jena, Germany) for 1 h in the dark at room temperature (RT). By SPAAC reaction the specific labeled CK2α-DBCO-Sulfo-Cy5 was obtained. For the site-specific labeling of CK2β^1-193^, purified CK2β^1-193^-pAzF in buffer P100 (25 mM Tris/HCl (pH 8.5), 100 mM NaCl) was treated with fluorescein alkyne (0.25 mM), TCEP (Tris(2-carboxyethyl)phosphine, 1 mM), TBTA (Tris(benzyltriazolylmethyl)amine, 0.17 mM) and CuSO_4_ (1 mM) for for 1 h in the dark at RT. By CuAAC reaction the specific labeled CK2β^1-193^-Flu was obtained. For MST measurements an additional ultrafiltration step using vivaspin500 columns (Sartorius, Göttingen, Germany) was used to remove unbound fluorophore and additives of the click reaction.

### 4.19. MST Measurements

For MST measurements CK2α-DBCO-Sulfo-Cy5 and CK2β^1-193^-Flu were applied to the Monolith NT.115 (NanoTemper Technologies GmbH, München, Germany). The concentration of the proteins were determined in triplicate by NanoPhotometer Pearl (Imlpen, München, Germany). For K_D_ value determination, 10 µL of CK2α-DBCO-Sulfo-Cy5 (100 nM) or CK2β^1-193^-Flu (100 nM) in kinase buffer (50 mM Tris/HCl (Ph 7.5), 25 mM NaCl, 20 mM MgCl_2_) supplemented with 0.1% Tween-20 were mixed with 10 µL B2 (50 mM Tris/HCl (pH 7.5), 25 mM NaCl, 20 mM MgCl_2_, 5% DMSO) in different concentrations. Fluorescence (red filter (CK2α-DBCO-Sulfo-Cy5), blue filter (CK2β^1-193^-Flu)) and thermophoresis (MST power 60%) were recorded at 37 °C for 30 s. The K_D_ value was determined from three independent measurements using MO.Affinity Analysis v2.1.3 software (NanoTemper Technologies GmbH).

## 5. Conclusions

Human protein kinase CK2 plays an important role in the genesis of cancer. Up to date several CK2 inhibitors were developed. Most of them target the highly conserved ATP cavity and show a weak selectivity throughout human protein kinases. Hence, there is increasing interest in the development of inhibitors with a different mode of inhibition. In the present study a potent peptidic non-ATP competitive inhibitor (B2) of CK2 holoenzyme and the α-subunit was identified with an IC_50_ value of 0.8 µM. At higher concentrations (≥ 25 µM) it disturbed also the interaction between the α-subunit and the β-subunit of CK2 to form the holoenzyme. No peptidic inhibitor of CK2 has been described before exhibiting these characteristics. B2 could serve as starting point in the design of new libraries in order to find a lead structure for the development of a new class of CK2 inhibitors. 

## Figures and Tables

**Table 1:** Inhibition of CK2 by peptides from bacterial surface display library screening.

Peptide	Sequence	Inhibition* (%)	IC_50_ Value (µM)
B1	KHTKGPTAYCPL	< 5	n.d.
B2	DCRGLIVMIKLH	79	0.8
B3	YRKPHWFIHTRI	< 5	n.d.
B4	PCPAPRAPKLSI	29	n.d.

n.d. = not determined, * 10 µM final concentration.
